# Differential production and secretion of potentially toxigenic extracellular proteins from hypervirulent *Aeromonas hydrophila* under biofilm and planktonic culture

**DOI:** 10.1186/s12866-020-02065-2

**Published:** 2021-01-06

**Authors:** Priscilla C. Barger, Mark R. Liles, Benjamin H. Beck, Joseph C. Newton

**Affiliations:** 1 Department of Biological Sciences, College of Sciences and Mathematics, Auburn University, Auburn, AL USA; 2 Biological Sciences, College of Sciences and Math, Auburn University, Auburn, AL USA; 3 USDA ARS Aquatic Animal Health Research Unit, Auburn, AL USA

**Keywords:** Hypervirulent *Aeromonas hydrophila*, vAh, Extracellular protein secretion, Secretome, Biofilm, Ecological niche adaptation, Pathogenicity, Virulence

## Abstract

**Background:**

Hypervirulent *Aeromonas hydrophila* (vAh) is an emerging pathogen in freshwater aquaculture that results in the loss of over 3 million pounds of marketable channel catfish, *Ictalurus punctatus*, and channel catfish hybrids (*I. punctatus, ♀ x* blue catfish*, I. furcatus, ♂*) each year from freshwater catfish production systems in Alabama, U.S.A. vAh isolates are clonal in nature and are genetically unique from, and significantly more virulent than, traditional *A. hydrophila* isolates from fish. Even with the increased virulence, natural infections cannot be reproduced in aquaria challenges making it difficult to determine modes of infection and the pathophysiology behind the devastating mortalities that are commonly observed. Despite the intimate connection between environmental adaptation and plastic response, the role of environmental adaption on vAh pathogenicity and virulence has not been previously explored. In this study, secreted proteins of vAh cultured as free-living planktonic cells and within a biofilm were compared to elucidate the role of biofilm growth on virulence.

**Results:**

Functional proteolytic assays found significantly increased degradative activity in biofilm secretomes; in contrast, planktonic secretomes had significantly increased hemolytic activity, suggesting higher toxigenic potential. Intramuscular injection challenges in a channel catfish model showed that in vitro degradative activity translated into in vivo tissue destruction. Identification of secreted proteins by HPLC-MS/MS revealed the presence of many putative virulence proteins under both growth conditions. Biofilm grown vAh produced higher levels of proteolytic enzymes and adhesins, whereas planktonically grown cells secreted higher levels of toxins, porins, and fimbrial proteins.

**Conclusions:**

This study is the first comparison of the secreted proteomes of vAh when grown in two distinct ecological niches. These data on the adaptive physiological response of vAh based on growth condition increase our understanding of how environmental niche partitioning could affect vAh pathogenicity and virulence. Increased secretion of colonization factors and degradative enzymes during biofilm growth and residency may increase bacterial attachment and host invasiveness, while increased secretion of hemolysins, porins, and other potential toxins under planktonic growth (or after host invasion) could result in increased host mortality. The results of this research underscore the need to use culture methods that more closely mimic natural ecological habitat growth to improve our understanding of vAh pathogenesis.

**Supplementary Information:**

The online version contains supplementary material available at 10.1186/s12866-020-02065-2.

## Background

*Aeromonas hydrophila* is a wide-spread and diverse species of Gram-negative bacterium ubiquitous in freshwater aquatic ecosystems. As a rapidly growing and metabolically diverse generalist [1–5], *A. hydrophila* is capable of exploiting a variety of ecological habitats and a broad range of hosts. *A. hydrophila* has been isolated from almost every freshwater aquatic environment and from diseased mammals, reptiles, amphibians, insects, and fish [1, 6–8]. *A. hydrophila* has been found in association with processed poultry, meats, fish, and even bottled water. It is capable of withstanding chlorination and is resistant to multiple antibiotics [1, 9].

In aquaculture, *A. hydrophila* is an important cause of disease in most freshwater production systems. Historically, *A. hydrophila* has been an important secondary pathogen in catfish production systems, commonly responsible for cutaneous ulceration and muscle necrosis. Occasionally following fish stress (low oxygen, poor water quality, etc.) the bacterium can cause a septicemia (motile aeromonad septicemia [MAS]), resulting in high mortalities [10–14]. In 2009, a new, highly virulent strain of *A. hydrophila* was isolated from a diseased channel catfish, *Ictalurus punctatus*, within a production pond in West Alabama. This strain, referred to as hypervirulent *Aeromonas hydrophila*, or vAh, was responsible for outbreaks of peracute motile aeromonad septicemia of epidemic proportions [11, 15–19]. vAh apparently acts as a primary pathogen, and may not be preceded by immune insult [11]. To date, vAh has been responsible for the loss of 30 million pounds of marketable channel catfish from production farms in West Alabama. In 2017, *A. hydrophila* infections were responsible for the loss of 3.4 million pounds of farm-raised catfish in Alabama alone, more than twice as much as the second leading cause of loss, *Flavobacterium columnare*. vAh has been the primary or secondary cause of catfish loss in Alabama since the primary outbreak in 2009 (Hemstreet, AL Fish Farming Center).

While much current research is focused on MAS disease prevention, there are many important unanswered areas of research to understanding the mechanisms of vAh pathogenesis, bacterial-host interactions, and bacterial adaptive responses under different environmental conditions. *A. hydrophila* are known to secrete a multitude of degradative and cytotoxic extracellular proteins which are widely accepted as virulence determinants [20–22], and which likely contribute to the environmental adaptability and broad species host range.

While vAh has established itself as a primary pathogen in natural settings [11], laboratory-cultured vAh appears to mimic its opportunistic relatives during immersion challenges. Planktonically-cultured vAh is extremely virulent, causing death in a matter of hours in intraperitoneal injection challenges. However, models meant to mimic more natural infections including submersion and gavage have been unreliable, even when challenged with artificially high colony forming units (CFUs) of the bacterium [2, 23]. Current studies of vAh pathogenesis and virulence are performed almost exclusively with planktonically-cultured bacteria despite the fact that most free-living generalist bacteria in aquatic systems reside primarily within biofilm [24–29]. One study using only planktonically-cultured vAh reported the presence of 228 extracellular proteins (ECPs) in the supernatant of vAh broth cultures, at least 23 of which were putative virulence factors [18], and a recent study comparing the secretomes of wild-type vAh with that of a group four capsule (gfc) - deficient vAh mutant reported the presence of multiple degradative and hemolytic proteins under planktonic culture [30]. A comparative proteomics study by Wang et al. [31] found differential expression of 33 proteins, many of which were involved in proteolysis, in response to iron starvation, underscoring the role of secreted proteins in environmental adaptation of vAh. Though niche adaptation clearly plays an important role in protein secretion, no studies have evaluated the secretome of biofilm grown vAh. A recent study by Cai et al. (2018) found no vAh present in the water column through the survey period, (July–October), while vAh resident in biofilm and pond sediment was detected at an increasing rate in the same sampling period, suggesting that biofilms serve as a stable reservoir for vAh survival when planktonic conditions are less favorable. Biofilm-associated bacteria generally have increased adhesive properties [24, 25, 32–34] and may have increased production of proteolytic enzymes, both of which could increase virulence [12, 35–37]. Redfield (2002) suggested that extracellular proteases are expressed when diffusion and/or mixing is reduced. vAh residing within a biofilm may have an advantage in attaching to and invading fish tissues due to increased secretion of proteolytic enzymes and adhesins. Given the data supporting the presence of vAh within pond biofilms, it is important to identify virulence factors secreted during biofilm residence that could impact host attachment and invasion. In this study, we compared the secreted protein profiles (secretomes) of biofilm- or planktonically cultured vAh strain ML09–119 to determine if niche occupation could influence vAh pathogenicity in natural environments.

## Results

### Biofilm grown vAh express higher protease activity but less hemolytic activity than planktonic cultures

**Fig. 1:**
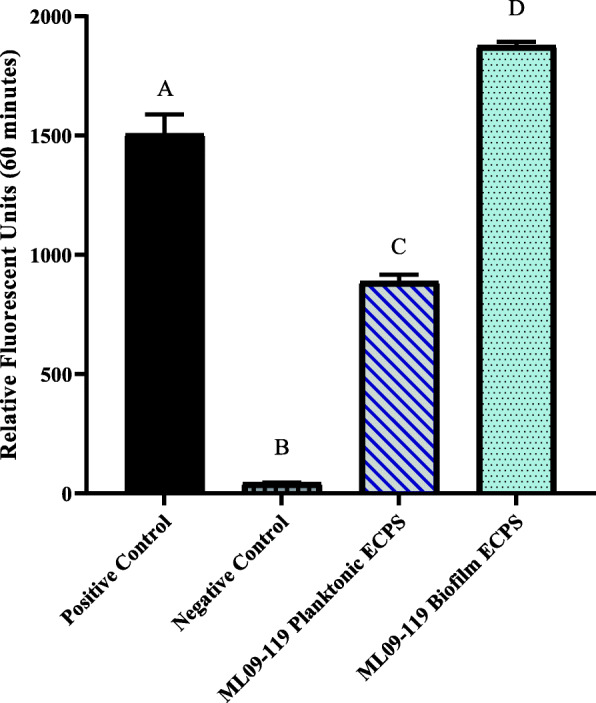
General proteolytic potential of vAh extracellular proteins (ECPs) secreted under biofilm and planktonic growth. The general proteolytic potential of biofilm and planktonic secretomes was measure using HiLyteFluor 488-labeled casein as a substrate. Secreted protein from each condition was incubated at 30 °C with labeled casein and fluorescent intensity was measured at Ex/Em = 490 nm/520 nm every five minutes for one hour. Data were plotted as relative fluorescence units versus time for each sample. Trypsin served as a positive control, and sterile, deionized water served as a negative control. Three individual experiments were performed, and all samples were performed in triplicate. Statistical analysis consisted of one-way ANOVA followed by Tukey’s multiple comparisons post-test with significance set at *p* < 0.05

**Fig. 2:**
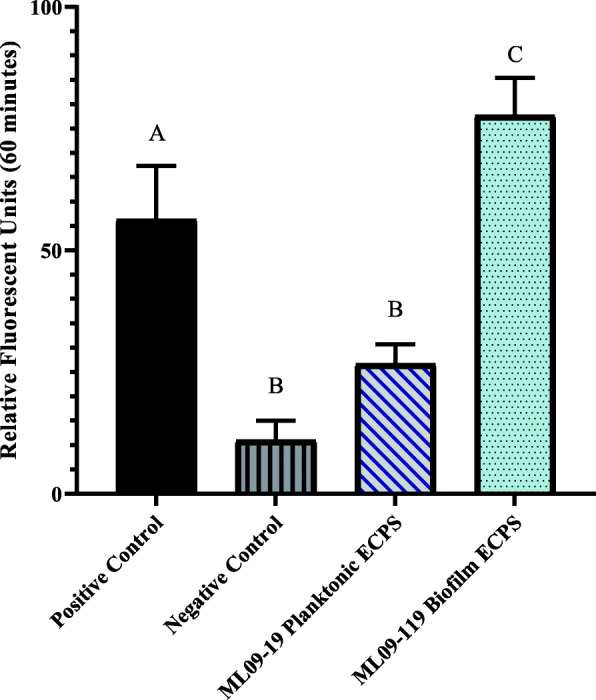
Elastase-specific degradative potential of vAh extracellular proteins (ECPs) secreted under biofilm and planktonic growth. The elastase activity of biofilm and planktonic secretomes was measure using 5-FAM/QXL™ 520-labeled elastin as a substrate. Secreted protein from each condition was incubated at 30 °C with labeled elastin and fluorescent intensity was measured at Ex/Em = 490 nm/520 nm every five minutes for one hour. Elastase served as a positive control and sterile, deionized water served as a negative control. Data were plotted as relative fluorescence units versus time for each sample. Three individual experiments were performed, and all samples were performed in triplicate. Statistical analysis consisted of one-way ANOVA followed by Tukey’s multiple comparisons post-test with significance set at *p* < 0.05

**Fig. 3:**
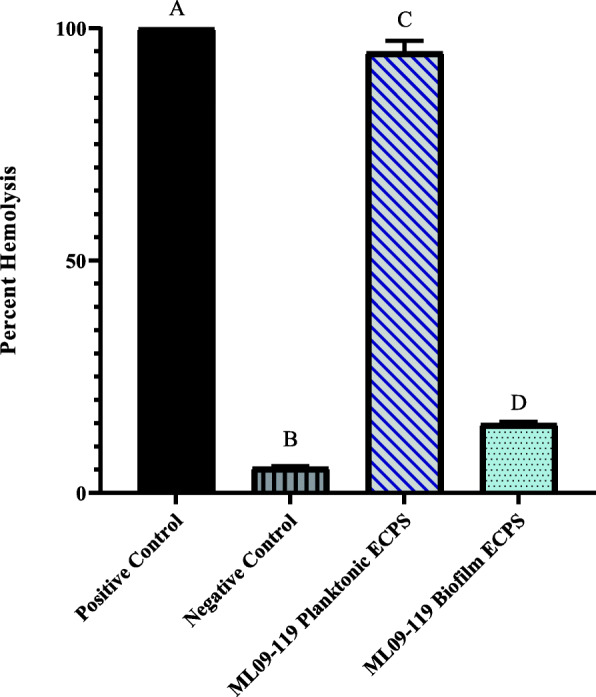
Hemolytic potential of vAh extracellular proteins secreted under biofilm and planktonic growth. The hemolytic ability of vAh secreted proteins was measured using channel catfish erythrocytes as the substrate. 2.5 μg secreted proteins from each culture condition was incubated with 25 μl catfish blood diluted 1:10 in sterile PBS. at 30 °C with shaking. Sterile, deionized water served as positive control and sterile PBS served as a negative control. Lysis was calculated by measuring sample absorbance at 415 nm, and reported as percent positive control. All samples were assayed in triplicate. Statistical analysis consisted of one-way ANOVA followed by Tukey’s multiple comparisons post-test with significance set at *p* < 0.05

Protease activity in biofilm samples was observed to be more than 2 times higher than in planktonic samples, and 1.2 times higher than the trypsin positive control (*p* < 0.05; Fig. 1). Similarly, elastase activity was significantly higher for biofilm grown vAh, which expressed 2.7 greater elastase activity than observed for planktonically grown cultures (*p* < 0.05; Fig. 2). In contrast, hemolytic activity was greatly increased in planktonic cultures, with more than 6 times higher hemolytic activity compared to biofilm grown vAh (*p* < 0.05; Fig. 3).

### Severe fish tissue necrosis was induced by the secretome of biofilm-grown vAh

**Fig. 4:**
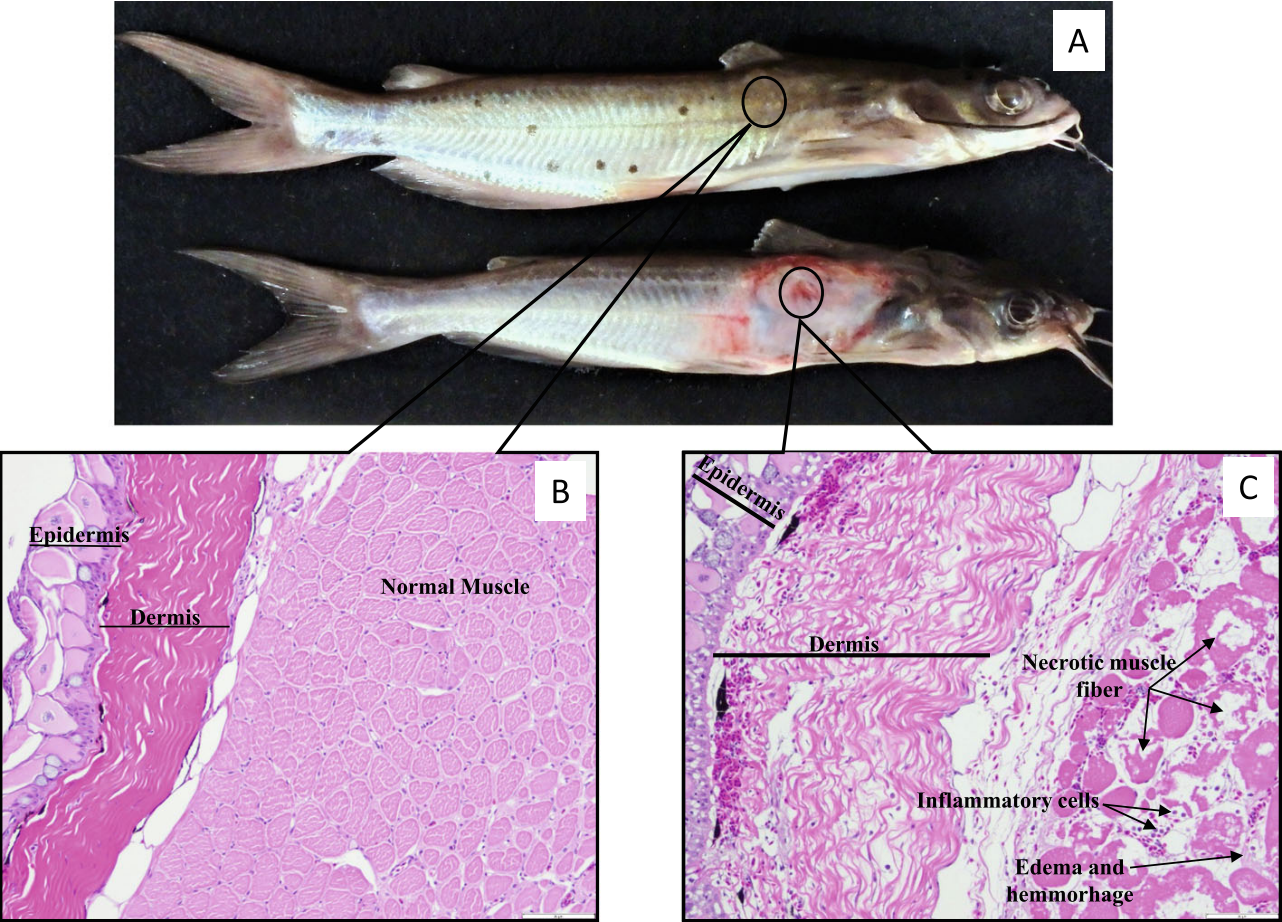
Gross lesions and histopathology of channel catfish muscle following intramuscular injection with vAh secreted proteins. (A) Channel catfish injected with Control (top) and biofilm-cultured vAh secreted proteins (bottom) 24 h post-injection, with black circle denoting injection site. Histologic sections prepared from paraffin-embedded tissues were stained with hematotoxylin and eosin. (B) Control (200X) – No perceptible damage to skin, subcutaneous adipose tissue, or muscle. Fish injected with planktonic ECPs were indistinguishable from controls. (C) Biofilm-injected fish tissue (200X) 24 h post-injection. Tissue was edematous, hemorrhagic, and necrotic at the injection site. Despite substantial tissue damage, few inflammatory cells were present

To determine if the increased proteinase and elastase activities observed from vAh when grown as an in vitro biofilm would result in tissue damage indicative of MAS disease, 10 μg of secreted proteins from each growth condition was injected intra-muscularly into channel catfish. Two hours post-injection, loss of dermal pigment was noted at the injection site in biofilm-injected fish, but no changes were observed for the injected planktonic secretome. After 24 h, substantial tissue necrosis was observed grossly in all biofilm-injected fish (Fig. 4). Fish injected with planktonic-associated ECPs developed no gross lesions even after 7 days. No control fish developed any gross lesion at the injection site after 7 days.

Histopathology was performed on skin and subcutaneous tissues collected from injection sites of channel catfish. Biofilm-injected fish tissue was edematous, hemorrhagic, and there was extensive tissue necrosis at the site of injection (Fig. 4). Despite substantial tissue damage, few inflammatory cells were present. In contrast, fish injected with planktonic ECPs were identical to the control fish, with no perceptible damage to skin, subcutaneous adipose tissue, or muscle.

### Biofilm and planktonically grown vAh have distinct secretomes

The differences in enzyme activities and tissue damage observed for biofilm versus planktonically-cultured vAh supported the hypothesis that niche occupancy has a significant influence on vAh exoprotein expression. To further test this hypothesis, a secretome analysis was performed to identify differentially secreted proteins present under the two culture conditions. A total of 272 proteins were identified in the secretomes of biofilm and planktonically-cultured vAh. Eighty-two proteins were identified that were present in both secretomes, while 98 were identified only in biofilm secretomes and 92 were unique to planktonic secretomes. ROTS and T-test analyses identified 53 proteins that significantly (FDR < 0.05, *p* = 0.01) varied in abundance. The protein abundance ratios of 52 ROTS-identified proteins were above the significant fold change threshold of ≥1.5 (Table 1). Thirty-five proteins were significantly increased in the biofilm secretomes, 20 of which were uniquely present in samples from biofilm grown vAh; for planktonic secretomes, 15 proteins were significantly increased in their abundance relative to biofilm samples, and these included nine proteins that were only observed from planktonic cultures. Of the proteins that varied significantly in their abundance, at least 15 from planktonic secretomes and 30 from biofilm secretomes have been indicated in virulence (Table 1, in bold font). However, not all secreted putative virulence proteins were differentially secreted, with many putative virulence factors identified in secretomes under both conditions.

**Table 1:** Differentially secreted proteins of planktonic and biofilm-cultured ML09–119

Secreted Protein	Locus Tag	ROTS-Statistic	*p* value	FDR	Protein Abundance Fold Change	Significant Experimental Group
Transcription regulation/ e- Transport						
**DNA gyrase inhibitor**	**AHML_21105**	2.2	0.01	0	16^a^	BIO
**Ribonuclease activity regulator**	**AHML_16315**	1.55	0.02	0.02	2.5	BIO
**FKBP-type peptidyl-prolyl cis-trans isomerase**	**AHML_05355**	−2.10	0.01	0	22^a^	TSB
Cytochrome d ubiquinol oxidase	AHML_19200	−1.71	0.02	0.02	16	TSB
Free Radical Scavenging						
**Superoxide dismutase**	**AHML_07590**	2.95	0.01	0	6	BIO
**Glyoxalase/dioxygenase protein**	**AHML_09045**	1.89	0.01	0.03	13^a^	BIO
Amino Acid/Cofactor Metabolism						
Redox protein (hypothetical)	AHML_05250	2.33	0.01	0	19^a^	BIO
**Diaminopimelate epimerase**	**AHML_02440**	1.82	0.02	0.03	9^a^	BIO
Ornithine carbamoyltransferase	AHML_21555	−1.35	0.03	0.05	3.5	TSB
Riboflavin-biosynthesis protein	AHML_17795	1.4	0.02	0.05	2.4	BIO
Urocanate hydratase	AHML_01870	3.71	0.00	0	8	BIO
Dihydrodipicolinate synthase	AHML_04540	2.35	0.01	0	22^a^	BIO
Succinylarginine dihydrolase	AHML_16715	5.49	0.00	0	66^a^	BIO
Carbohydrate Metabolism						
**Maltose operon periplasmic protein**	**AHML_06220**	2.57	0.01	0	15^a^	BIO
**Phosphoglyceromutase**	**AHML_01445**	1.94	0.01	0	13^a^	BIO
**Transaldolase B**	**AHML_16890**	8.08	0.00	0	89^a^	BIO
**Pullulanase**	**AHML_04415**	3.62	0.00	0	10	BIO
**Ribose-5-phosphate isomerase A**	**AHML_14480**	2.92	0.01	0	3	BIO
**Beta-glucosidase**	**AHML_14270**	−1.68	0.02	0.0	14	TSB
Outer Membrane Proteins						
**Outer membrane protein A**	**AHML_21905**	−1.99	0.01	0	4	TSB
**Outer membrane protein A**	**AHML_20145**	1.46	0.02	0.04	13^a^	BIO
**Outer membrane lipoprotein**	**AHML_00700**	−1.58	0.02	0.02	13^a^	TSB
Degradative Enzymes and Toxins						
**Hemolysin (Aerolysin-type)**	**AHML_02265**	−2.37	0.01	0	3	TSB
**Hemolysin (ahh1-type)**	**AHML_08400**	−2.69	0.01	0	4	TSB
**Elastase**	**AHML_04340**	4.68	0.00	0	5	BIO
**Chitinase**	**AHML_05225**	4.07	0.00	0	3	BIO
**Metalloprotease**	**AHML_05230**	2.73	0.01	0	3	BIO
**Basic endochitinase**	**AHML_05235**	3.15	0.01	0	3	BIO
**Zn-dependent carboxypeptidase**	**AHML_05535**	2.15	0.01	0	13^a^	BIO
**Outer membrane porin protein**	**AHML_04355**	2.91	0.01	0	15^a^	BIO
**Extracellular lipase**	**AHML_00550**	−4.16	0.01	0	3	TSB
**Chitin-binding domain 3**	**AHML_11110**	−1.50	0.02	0.02	12	TSB
**Zn-dependent protease with chaperone function**	**AHML_06635**	1.51	0.02	0.02	11^a^	BIO
**Phosphoglyceromutase**	**AHML_01445**	1.94	0.01	0	13^a^	BIO
**Phospholipid-cholesterol acyltransferase**	**AHML_20135**	−1.55	0.02	0.02	14^a^	TSB
**Aminopeptidase**	**AHML_18480**	−1.55	0.02	0.02	2	TSB
**Chitin binding protein**	**AHML_03125**	1.59	0.02	0.02	1.6	BIO
**Proline iminopeptidase**	**AHML_18440**	1.88	0.01	0.03	14^a^	BIO
**Serine Protease**	**AHML_14260**	−1.91	0.01	0.03	19	TSB
**Periplasmic carboxy-terminal protease**	**AHML_11480**	2.28	0.01	0	14^a^	BIO
**Collagenase family**	**AHML_02655**	−1.90	0.01	0.03	2.5	TSB
**Extracellular alkaline serine protease**	**AHML_18455**	−2.22	0.01	0	25^a^	TSB
**Small protease**	**AHML_08855**	1.37	0.03	0.05	8^a^	BIO
Pilus and Flagellin Proteins						
**Flagellin**	**AHML_09350**	5.42	0.00	0	43^a^	BIO
**Flagellin-like protein**	**AHML_09345**	2.05	0.01	0	2.6	BIO
**Type I pilus assembly protein FimF**	**AHML_02690**	−2.29	0.01	0	26^a^	TSB
**Fimbrial Proteain**	**AHML_02665**	−2.61	0.01	0	36^a^	TSB
Transport Proteins						
**ABC-type sugar transport**	**AHML_20895**	1.77	0.02	0.02	14^a^	BIO
**TonB-dependent copper receptor**	**AHML_02545**	1.79	0.02	0.02	9	BIO
**Peptide ABC transporter**	**AHML_17755**	3.2	0.00	0	98^a^	BIO
**Arginine ABC transporter**	**AHML_03370**	4.58	0.00	0	7	BIO
**Oligopeptide ABC transporter**	**AHML_13875**	6.05	0.00	0	93^a^	BIO
**Leucine binding protein (ABC transport)**	**AHML_00595**	3.74	0.00	0	18	BIO

Differentially secreted proteins of vAh ML09–119 cultured planktonically (TSB) and within a biofilm (BIO). Proteins are grouped based on their major biological process, determined by gene ontology. Protein abundance fold change marked with ^a^ denotes protein identified in only one condition and is reported as the average Quantitative Protein Value. Proteins and locus tags in **Bold** indicate putative virulence proteins. FDR = False Discovery rate

**Fig. 5:**
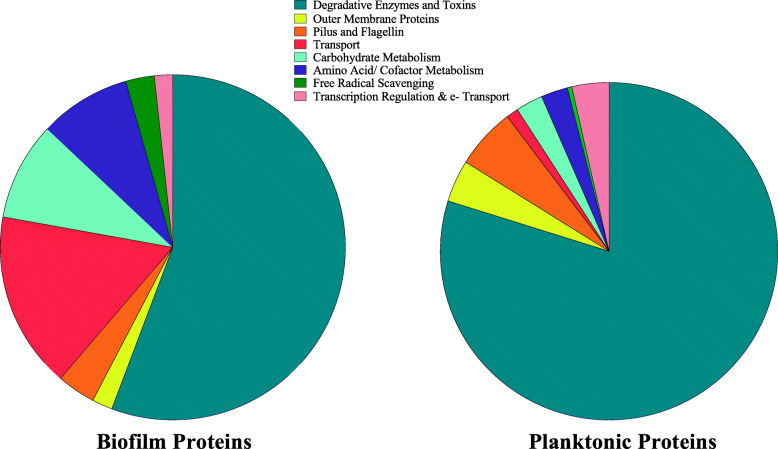
Functional categories of differentially secreted proteins from vAh cultured planktonically or within a biofilm. Primary biological function was assessed by gene ontology analyses. Proteins were grouped into eight functional categories based on their gene ontology annotation and plotted as part-of-a-whole. In both biofilm and planktonic secretomes, differentially secreted proteins were dominated by degradative enzymes and toxins

Functional group comparisons based on gene ontology (Table 1, Fig. 5) revealed extensive secretion of degradative enzymes and toxins in both biofilm and planktonic secretomes, with degradative enzymes, such as elastase, metalloprotease, chitinase, and endochitinase, dominating biofilm secretomes and cytotoxic and cytotonic toxins, such as *ahh1*-type hemolysin and extracellular lipase enriched in planktonic secretomes. In both planktonic and biofilm secretomes, degradative enzymes and toxins made up the majority of significant proteins, representing 79.8% of planktonic proteins and 55.7% of biofilm proteins. Proteins involved in transport (16.5%), carbohydrate metabolism (8.5%), and pilus and flagellin (3.6%) contributed significantly to the biofilm secretome, while pilus and flagellin proteins (5.8%), outer membrane proteins (4.0%), and proteins involved in transcriptional regulation and electron transport (3.5%) were other significant contributors to planktonic secretomes (Fig. 5). Of particular interest were the presence of polar flagellar proteins (*AHML_09345* and *_09350*) present in higher quantities in the biofilm secretome and type I pili proteins (*AHML_2665* and *_2690*) that were present in planktonic secretomes, but absent from biofilm secretomes. Polar flagella, typically considered motility flagella, are important in adhesion and invasion in *A. hydrophila* that lack lateral flagella, such as vAh [38], while type I pili are thought to contribute to host colonization, but not host invasion [39].

## Discussion

While vAh are significantly more virulent that traditional *A. hydrophila* when challenged by intraperitoneal injection [15], Zhang et al. [40] reported consistent vMAS mortality was attainable in channel catfish immersion trials only following scarification and challenge with 2 × 10^7^ CFU/ml of planktonically-cultured vAh. This suggests that some environmental stimuli are not present in artificial broth culture, which, in pond systems, could be responsible for inducing bacterial virulence and resulting in large scale MAS epidemic outbreaks. Since most environmental bacteria spend much of their time in biofilm, either attached to a substrate or floating as bacterial flocs [24, 26, 27, 29, 41], biofilm-associated vAh may produce proteins that increase invasiveness and allow initial colonization in vivo [42]. The ability to form a biofilm is commonly considered a virulence factor, particularly in human disease conditions [43]. Likewise, for *A. hydrophila* biofilm formation and residency may induce global changes in gene expression resulting in increased production and secretion of degradative enzymes and other factors that increase pathogenicity or invasiveness. *Aeromonas* spp*.* produce extracellular enzymes that facilitate nutrient acquisition in aquatic environments and produce adhesins that aid in the attachment and colonization of benthic surfaces [5]. In aquatic environments, these enzymes provide nutrients by degrading the organic compounds including suspended detritus and benthic substrates. These enzymes may also be important in the pathophysiology of disease by enabling degradation of animal tissues [5, 24, 44].

Previous research reported the presence multiple potentially toxigenic extracellular proteins in the supernatant of planktonically-cultured vAh [18, 30]. Because many opportunistic bacteria like vAh reside largely in biofilms and not as sustained planktonic populations [45], it was important to evaluate the influence of biofilm growth on vAh exoprotein expression. This study found that degradative activities were significantly increased in the supernatant of biofilm-associated vAh (Figs. 1 and 2). Furthermore, when biofilm-grown vAh ECPs were injected into the muscle of channel catfish, significant necrosis and cytolysis occurred within 24 h, while secreted proteins of planktonically-cultured vAh failed to produce necrotic lesions after seven days.

A secretome analysis was conducted to examine in more detail how biofilm growth influenced vAh exoprotein expression, which revealed significant differences in the secretomes of the two cultures, both in complexity and quantity. The biofilm secretome contained 248 proteins, including 183 unique proteins, while planktonic secretomes contained 183 total proteins, including 101 unique proteins. Of the 82 proteins secreted under both culture conditions, at least 36 had previously been identified as putative virulence factors [18, 21, 46]. Under both growth conditions, vAh secreted an abundance of potential virulence proteins, the majority of which were not statistically significant in differential secretion analyses. However, secretomes of vAh cultured in biofilm were significantly more varied and, in general, relative protein abundance was increased.

Assays to measure general and specific proteolytic potential of the secreted proteins revealed significant increases in both caseinolytic and elastinolytic activity in biofilm secretomes when compared to planktonic ECPs (Figs. 1 and 2). A significant difference in proteolytic potential was also seen upon inspection of the secretome analysis. ROTS analysis revealed at least seven degradative proteins were present in the biofilm secretomes at significantly higher observed abundance relative to planktonic secretomes. There was a 5-fold increase in elastase abundance in biofilm secretomes, with an average quantitative protein value (QPV) of 122, compared to an average QPV of 23 in planktonic secretomes, which agreed with the results obtained from elastase enzyme activity measurements. There was a 3-fold increase in the M66 - family metalloprotease *AHML*_*05230* in biofilm secretomes, with average QPVs of 103 and 30 in biofilm and planktonic secretomes, respectively. Both elastase and the M66 zinc metalloprotease are considered significant virulence factors of *A. hydrophila* as well as other pathogens, such as *Vibrio cholerae*, and enterohemorrhagic *Escherichia coli* [47, 48]. Five other proteolytic enzymes were secreted in statistically significant quantities in biofilm secretomes but were not detected in the planktonic secretomes and likely increase the overall proteolytic potential of biofilm ECPs (Table 1).

While the majority of the differentially secreted degradative enzymes present in the biofilm secretome were proteolytic, two important glycolytic proteins, chitinase and chitin binding protein (CBP) were found in significantly higher amounts in biofilm secretomes. While chitinase and CBP are integral in the breakdown of environmental chitin, these proteins may also play integral roles in virulence. Though vAh can use chitin as a sole carbon source [49], the lack of chitin in the TSB growth medium would make it unlikely that chitinase and CBP production would be energetically favorable. Therefore, it is hypothesized that these chitin-associated proteins play other roles in vAh fitness or pathogenicity. In other pathogens, chitinases and CBPs are considered virulence factors not because they target chitin but because of their interactions with substrates other than chitin. In some virulent *E. coli* and *V. cholerae*, chitinases and CBPs target host glycoproteins and glycolipids that contain N-acetylglucosamine (GlcNAc), the monomer present in mucus [50, 51]. Outer membrane-expressed chitinases and CBPs have also been indicated as accessory molecules responsible for initiating host cell adhesion and invasion [50–52]. In a murine model, *E. coli* chitin-binding domain interacts with intestinal epithelial cells, increasing invasiveness and pathogenicity [51]. In *V. cholerae*, Bhowmick et al. (2008) found chitinases function to break down the GlcNAc of mucin and reported upregulation of chitinases resulting from exposure to exogenous mucin. Furthermore, the *V. cholerae* chitin binding protein GbpA was shown to bind to the protective mucus layer of mammalian intestinal epithelium, resulting in bacterial colonization and disease initiation. Likewise, chitinases and CBPs produced by clinical *Pseudomonas aeruginosa* strains isolated from patients with cystic fibrosis (CF) were also upregulated in response to mucin-containing sputum and likely play an integral role in primary adhesion to lung epithelium in the initiation of CF [50]. In fish, the mucosal barrier covering the gills, skin, and intestinal surfaces are considered the first line of defense against invading pathogens [53, 54]. The presence of chitinases and CBP may act to degrade not only the catfish slime coat, but also to bind to and degrade the epithelial mucins in the digestive tract, increasing vAh invasiveness. Peatman et al. (2018) reported a direct link between feed consumption and vAh-induced MAS, with survival in vAh-challenged catfish decreasing significantly when fish were fed to satiation 4 h prior to challenge. The mucus coating of the intestinal epithelium may decrease after eating, as ingesta moves through the digestive tract and takes mucus with it. Chitinases and CBPs may then be capable of breaking down the remaining mucus, gaining access to the underlying epithelium and, eventually, the bloodstream [55]. The presence of chitinase and CBP could help explain the intestinal epithelial damage found on necropsy in fish naturally infected with vMAS [56]. Although significantly higher in biofilm secretomes, chitinase and CBP was prominent in both planktonic and biofilm secretomes, suggesting they play an important role in bacterial fitness regardless of growth condition.

Whereas biofilm secretomes were flush with degradative exoenzymes, such as elastase, chitinases, and multiple Zn-dependent and metalloproteases, planktonic secretomes consistently produced more hemolytic and cytotoxic ECPs. Notably, both aerolysin-type and *ahh1*-type hemolysins were detected in much higher quantities in planktonic secretomes, as were two extracellular serine proteases (neither of which were identified in any biofilm sample) and extracellular lipases, all of which exert hemolytic activity against erythrocytes, and have been shown to be cytotoxic to cells [20, 57]. Interestingly, the alpha-hemolysin, phospholipid-cholesterol acyltransferase, which was present in planktonic secretomes but absent in biofilm, has been reported to produce significant lysis of salmon erythrocytes following activation by serine protease [58]. The presence of substantial amounts of both proteins in the planktonic secretomes suggests that the production of these proteins could allow a multi-pronged approach to cell death, with each toxin acting independently, but increasing the collective virulence resulting from multiple exoproteins. Aerolysin-type hemolysin has been implicated as the main virulence factor of *A. hydrophila* [20], and was significantly higher in planktonic secretomes, with a three-fold increase compared to biofilm. However, ahh1-type hemolysin was present in planktonic secretomes at greater than three times the amount of aerolysin-type hemolysin. Ahh1 hemolysins are homologous to hlyA hemolysins of *V. cholerae* [59]. The activity of this pore-forming hemolysin is not erythrocyte-specific, but targets erythrocytes, leukocytes, lymphocytes, and epithelial and endothelial cells in a multitude of eukaryotes [60] and, as such, are considered cytotoxins. This supports the in vitro hemolysis assay results that found 80% hemolysis of channel catfish erythrocytes in one hour when exposed to planktonic supernatants, compared to less than 15% average hemolysis of erythrocytes that were incubated with biofilm supernatants (Fig. 3). The presence of these hemolysins and other cytotoxins in planktonically-cultured vAh may also help explain the rapid mortality seen in catfish when challenged by intraperitoneal injection, as these bacteria may be primed to produce vast amounts of toxins in vivo.

Biological functions of secreted proteins as analyzed by gene ontology found carbohydrate utilization to be the dominant function of secreted proteins under both conditions. Proteins involved in hemolysis, lipid and nucleotide catabolism, arginine biosynthesis, protein folding and transport were dominant biological functions of planktonic secretomes. Significant biofilm proteins were largely involved in transmembrane transport, amino acid processing, and transport of ions, amino acids, and carbohydrates. Interestingly, flagellar motility was also important in biofilms. This is likely due to *A. hydrophila*’s use of flagella in biofilm construction and not for bacterial motility [38]. This increase in polar flagella may also contribute to an increased host colonization in biofilm-associated vAh. While lateral flagella are often considered imperative for biofilm production and adhesion [33, 61], Aeromonads that lack lateral flagella are capable of using polar flagella for biofilm formation as well as cellular adhesion [34, 38, 62, 63]. The increased polar flagella required for biofilm formation could act secondarily as adhesins when biofilm-derived bacteria come into contact with catfish mucosal surfaces and could act in concert with other secreted invasins to colonize and destroy host mucosal barriers.

## Conclusions

Most aquatic bacterial generalists, such as *A, hydrophila*, spend the majority of time resident in biofilms and host-microbe interactions are likely influenced by niche-specific microbial phenotype. Because biofilm-associated bacteria have emergent properties that cannot be elucidated by the study of free-living cells, it is imperative to study organisms within biofilms to understand how niche adaptations may influence overall pathogenicity and virulence. This study is the first comparison of the secreted proteomes of vAh when grown in two distinct ecological niches. These data on the adaptive physiological response of vAh based on growth condition increase our understanding of how environmental niche partitioning could affect vAh pathogenicity and virulence. Increased secretion of colonization factors and degradative enzymes during biofilm growth and residency may increase bacterial attachment and host invasiveness, while increased secretion of hemolysins, porins, and other potential toxins under planktonic growth (or after host invasion) could result in increased host mortality. These shifts in protein expression and secretion indicate that growth under biofilm and planktonic conditions results in massive changes in gene expression. Future research should explore the global regulatory factors that affect vAh gene expression under these growth conditions. Taken together, these data may help in our understanding of the unique aspects of this emerging pathogen that contribute to the devastating impact of MAS disease outbreaks.

## Methods and materials

### Bacterial strain

vAh strain ML09–119 was isolated from a diseased channel catfish from a MAS outbreak in a West Alabama aquaculture facility in 2009. Molecular characterization and genome sequencing of vAh ML09–119 have been performed [19] and the complete genome sequence deposited in GenBank (Accession CP005966). Aliquots of vAh ML09–119 were cryogenically stored in 10% glycerol freeze medium at − 80 °C.

#### Catfish

Specific-pathogen free channel catfish fingerlings maintained under Auburn University IACUC-approved protocol 2018–3251 (Catfish Production and Maintenance) were used for challenges. All challenges were performed adhering to the guidelines of AU-IACUC-approved protocol 2016–2900 (Identification of toxigenic proteins of virulent *Aeromonas hydrophila* and evaluation of host response).

### Culture media and culture conditions

Tryptic soy broth (TSB) (Bacto TSB, BD) prepared according to manufacturer’s directions was used as the culture medium for planktonic growth.

Biofilm media was prepared by adding 0.2% agar powder (AlfaAesar) to TSB media prior to sterilization, and 70 ml of molten agar was poured into deep well petri dishes (Fisher), as previously described [64].. An aliquot of vAh ML09–119 was removed from cryogenic storage and cultured in TSB overnight at 30 °C on an orbital shaker. This culture was then used to prepare planktonic and biofilm cultures, as previously described [64]. Briefly, 70 ml of fresh TSB media was inoculated with 1 ml of the overnight culture and grown at 30 °C with shaking to mid-log phase. Biofilm agar plates were inoculated from overnight culture by stab inoculation, sealed with parafilm, and incubated at 30 °C for 72 h. Planktonic and biofilm cultures were performed in triplicate.

### Secretome preparation

#### Planktonic Secretome

Secreted proteins of planktonically-cultured vAh ML09–119 were purified from cell-free supernatants, prepared as previously described [64]. Briefly, vAh was cultured in TSB media as described above, cells were pelleted by centrifugation, and supernatant was decanted and retained. Pelleted cells were washed twice with cold, sterile PBS, pelleted as above, and the wash was added to the supernatant. Combined supernatants were then passed through a low-binding 0.22 μm vacuum filter (VWR) to remove any remaining cells.

#### Biofilm Secretome

Secreted proteins of biofilm-cultured vAh ML09–119 were purified from cell-free supernatants, as previously described [64]. In brief, cells were removed from the biofilm media surface and washed twice with cold, sterile PBS as described above. The cell wash was decanted and retained. Secreted proteins were then collected from within the biofilm media by disrupting the biofilm media until the soft agar had formed a slurry. The slurry was transferred to conical tubes, centrifuged to pellet the agar, and the liquid media was decanted and retained. The agar plug was then resuspended in sterile PBS and centrifuged as above. The wash solutions was decanted and retained. All retained supernatants were combined and residual agar and bacterial cells were removed by filtration through a low-binding 0.45 μm and 0.22 μm vacuum filters (VWR).

#### Ammonium sulfate precipitation

Extracellular proteins (ECPs) were precipitated from cell-free supernatants by ammonium sulfate precipitation, as previously described [30, 64]. Briefly, ammonium sulfate crystals (Fisher Scientific) were added to cell-free supernatants to achieve 65% saturation and incubated at 4 °C with gentle mixing for 24 h. Precipitated proteins were collected by centrifugation, resuspended in Tris buffer, and dialyzed twice against the same buffer in 10 Kda dialysis cassettes (Slide-A-Lyzer (Thermo Fisher)). Following dialysis, the volume of all protein samples were adjusted to 20 ml by the addition of cold Tris buffer. Protein concentration of each sample was determined by the Bradford assay (Pierce Coomassie Plus Protein Assay, Thermo Fisher). These concentrated proteins were used for all enzymatic assays.

### Enzymatic activity

The in vitro activity of secreted proteins was measured using specific substrates to determine the degradative and toxigenic potential of planktonic and biofilm secretomes, as described below:

#### Hemolysis

Hemolytic potential was measured using the method of Barger et al. (2020). In brief, heparinized blood from channel catfish was diluted 1:10 in sterile PBS and incubated with a suitable dilutions of protein for 2 h at 30 °C in an orbital shaker. Positive control tubes representing 100% hemolysis contained sterile distilled water in place of protein samples. Negative control tubes contained sterile PBS in place of protein samples. Following incubation, tubes were centrifuged to pellet un-lysed erythrocytes and supernatant was transferred to clear, 96-well flat bottom plates. Hemolysis was quantified by measuring absorbance of released hemoglobin at 415 nm in multi-mode plate reader (Synergy HTX, Bio-Tek) and hemolysis was reported as percent of positive control.

#### Universal protease activity

Non-specific proteolytic activity was measured using HiLyteFluor 488-labeled casein as the substrate, following manufacturer’s protocol with minor modifications (Sensolyte Green Fluorimetric Protease Assay Kit, AnaSpec, Inc.), as previously described [64]. Briefly, a suitable concentration of protein was added to triplicate wells of black, flat-bottom 96-well plates with non-binding surface (Greiner Bio-One). Trypsin served as a positive control and sterile deionized water served as a substrate control. Labeled casein was added to each well and relative fluorescence was measured at Ex/Em = 490 nm/520 nm every five minutes for one hour in a multi-mode plate reader (Synergy HTX, Bio-Tek) with 30 °C incubation temperature. Data were plotted as relative fluorescence units versus time for each sample.

#### Elastase activity

Elastase-specific activity was measured using 5-FAM/QXL™ 520 labelled elastin as the substrate, following the manufacturer’s protocol with minor modifications (Sensolyte Green Fluorimetric Elastase Assay Kit, AnaSpec, Inc.), as previously described [64]. Briefly, a suitable concentration of protein was added to triplicate wells of black, flat-bottom 96-well plates with non-binding surface. Positive and negative controls were elastase and sterile, deionized water, respectively. Labeled elastin substrate was then added to each well and relative fluorescence was measured continuously at Ex/Em = 490 nm/520 nm for one hour in a multi-mode plate reader (Synergy HTX, Bio-Tek) with 30 °C incubation temperature. Data were plotted as relative fluorescence units versus time for each sample.

### In vivo proteolysis

Extracellular protein activity was measured in vivo using channel catfish fingerlings to determine potential proteolytic and cytotoxic tissue effects.

#### Protein preparation

Ten microgram aliquots of secreted planktonic and biofilm-associated proteins, prepared as above, diluted in 100 μl sterile PBS were used for injection challenges.

#### Challenge model

Channel catfish fingerlings were transferred to 57-l glass aquaria containing dechlorinated municipal water and acclimated at 30 °C for two days prior to challenge. Triplicate tanks containing five fish each represented planktonic ECP, biofilm-associated ECP, and injection control groups. Prior to injection, fingerlings were transferred to sedation aquaria containing 70 mg/ L tricaine methanesulfonate (MS-222) buffered to neutrality with sodium bicarbonate. Following sedation, characterized by decreased opercular movement and loss of equilibrium, 100 μl of sterile PBS containing 10 μg of total protein was injected intramuscularly just below the dorsal fin using tuberculin syringes fitted with 26 gauge needles. Control fish were injected with 100 μl sterile PBS. Fish were then returned to the appropriate aquarium and monitored until fully recovered. Fish were maintained in aquaria at 30 °C for 7 days under flow-through conditions at 1 gal per hour water replacement. Moribund fish or fish developing severe external lesions were euthanized by prolonged exposure to buffered MS-222, the tissues were collected and fixed in 10% neutral-buffered formalin. After 7 days, remaining fish were humanely euthanized and samples were collected and prepared as above.

#### Histology

Formalin-fixed tissues were paraffin-embedded and 4 μm sections were prepared and stained with hematoxylin and eosin according to standard methods [65]. Slides were evaluated and photographed using an Olympus BX53 microscope with an Olympus UPlanFL N 20X/0.50 objective, fitted with an Olympus DP26 digital camera, and captured with Olympus cellSens Entry Imagining software (Olympus Corporation). No further imaging processing or manipulation was performed on photomicrographs.

### Secretome analysis

To determine how vAh niche occupancy might influence protein production, secreted protein profiles of vAh cultured within a biofilm and in broth were compared by liquid chromatography with tandem mass spectrometry (LC MS/MS) analysis at the University of Alabama at Birmingham Mass Spectrometry/Proteomics shared facility (Birmingham, Alabama, USA) to identify and quantify proteins present in each sample, as previously described [30], as follows.

#### Proteomics analysis

Samples were prepared for analysis as follows: 20 μg of protein per sample in NuPAGE LDS sample buffer (Invitrogen) was loaded onto a Novex NuPage 10% Bis-Tris protein gel (Invitrogen), separated as a short stack, and stained overnight with Novex Colloidal Blue Staining kit (Invitrogen). Gels were then destained and lanes were cut into single molecular weight fractions. Following equilibration in 100 mM ammonium bicarbonate, fractions were digested overnight with Trypsin Gold (Mass Spectrometry grade (Promega)) and peptide extracts were reconstituted in 0.1% formic acid to 0.1 μg/μl.

#### Mass spectrometry

Digested samples were analyzed on a 260 Infinity HPLC stack (Agilent Technologies) and chromatographic separation occurred on a C18 reverse-phase column (Jupiter C-18, 71 μ × 15 cm, 300 Å, 5 μm (Phenomenex)) with an in-line Thermo Orbitrap Velos Pro hybrid mass spectrometer, equipped with a nano-electrospray source (Thermo Fisher). Binary mobile phase solvents were comprised of 0.1% formic acid (solvent A) and 0.1% formic acid in 85% acetonitrile (solvent B). All data were collected in CID mode. A parent scan range of 300 to 1200 m/z (at 60 K resolution) was chosen and fragmentation data (MS2) were collected on the top 15 most intense ions. For data-dependent acquisition, charge-state screening and dynamic exclusion were enabled with a repeat count of 2, repeat duration of 30s, and exclusion duration of 90s.

#### Mass spectrometry data conversion and searches

Data acquisition was executed using Xcalibur software. Xcalibur RAW files were collected in profile mode, converted to centroid data, and then converted to mzXML using ReAdW v3.5.1 (IonSource). The mgf files were then created using MzXML2Search (included in Trans-Proteomics Pipeline v3.5) for all scans. The data were searched with a species-specific subset of the UniRef 100 database using SEQUEST (Thermo Fisher, San Jose, CA, USA; version 27), which was set for two maximum missed cleavages, a precursor mass window of 20 ppm, trypsin digestion, variable modification C at 57.0293, and M at 15.9949.

#### Peptide filtering, grouping, quantification and statistical analyses

Scaffold (v. 4.8.4, Proteome Software Inc., Portland, Oregon) was used to validate MS/MS based peptide and protein identifications. Peptides identified by SEQUEST search were filtered with Scaffold. A minimum peptide length of > 5 amino acids, with no MH+ charge states, peptide probabilities of > 80% C.I., and with the number of unique peptides per protein ≥2 were set as filter cut-off values required to accept peptide identification. Peptide probabilities were assigned by the PeptideProphet algorithm [66, 67]. The two most common methods for statistical validation of large proteome data, False discovery rate (FDR) and protein probability, are incorporated in Scaffold. Protein identifications were accepted if proteins probabilities could be established at > 99% C.I., contained at least 4 identified peptides, and with false discovery rate < 1.0. Spectral counting and was performed for relative quantification across samples. Spectral count abundances were normalized between samples, when relevant. Proteins present in at least two experimental replicates were included in analyses. To identify differentially secreted proteins, two nonparametric statistical analyses including reproducibility-optimized test statistic (ROTS) (bootstrapping value = 1000) combined with single-tail t-test (*p* < 0.05) [68, 69] were performed between each pair-wise comparison. These were then sorted according to the highest statistical relevance in each comparison. For protein abundance ratios determined by normalized spectral counts, a fold change threshold ≥1.5 was set for significance. For proteins present in only one experimental group, the average of the normalized quantitative value was designated as the protein abundance.

#### Protein function

To define the potential function of secreted proteins, major biological processes of statistically significant proteins were determined from gene ontology annotation in UniProt (Consortium, T.U. 2018) and QuickGO [70]. Predicted protein function was assessed by determining major biological processes through gene ontology. Using these data, eight functional groups were established, and proteins were sorted into these groups based on their primary biological function. A further comparison was made by compiling all proteins in each functional group from both biofilm and planktonic secretomes and expressing as parts of a whole, with side-by-side comparisons for each secretome type.

### Statistical analyses

Reproducibility-optimized test statistic (ROTS) analysis of differentially secreted proteins was performed in R [71]. All other statistical analyses were performed in Prism 8.2.0 (Graphpad). One-way ANOVA followed by Tukey’s multiple comparisons post-test were performed on triplicate data with significance set at *p* < 0.05. Graphical representations of data were produced in Prism 8.2.0.

## Supplementary Information

**Additional file 1.**

**Additional file 2.**

## Data Availability

Data generated or analyzed during the current study are included in this published article and its supplementary information file.
