# Correction to: Differential production and secretion of potentially toxigenic extracellular proteins from hypervirulent *Aeromonas hydrophila* under biofilm and planktonic culture

**DOI:** 10.1186/s12866-021-02088-3

**Published:** 2021-01-22

**Authors:** Priscilla C. Barger, Mark R. Liles, Benjamin H. Beck, Joseph C. Newton

**Affiliations:** 1 Department of Pathobiology, College of Veterinary Medicine, Auburn University, Auburn, AL USA; 2 Biological Sciences, College of Sciences and Math, Auburn University, Auburn, AL USA; 3 USDA ARS Aquatic Animal Health Research Unit, Auburn, AL USA

**Correction to: BMC Microbiol 21, 8 (2021)**

**https://doi.org/10.1186/s12866-020-02065-2**

Following publication of the original article [1], we were notified of a mistake in author affiliation 1, which was introduced after the proofs were approved.

Originally published affiliation: Department of Biological Sciences, College of Sciences and Mathematics, Auburn University, Auburn, AL, USA.

Corrected affiliation: Department of Pathobiology, College of Veterinary Medicine, Auburn University, Auburn, AL, USA.

The original article has been corrected.

The publisher apologizes for any inconvenience.
