# Supplementary material for: Differential production and secretion of potentially toxigenic extracellular proteins from hypervirulent Aeromonas hydrophila under biofilm and planktonic culture
Source: BMC Microbiol. 2021 Jan 6;21:8. doi: 10.1186/s12866-020-02065-2 (PMC7788984; doi:10.1186/s12866-020-02065-2)
Supplement: Supplementary file 2 — Additional file 2. [file 12866_2020_2065_MOESM2_ESM.docx]

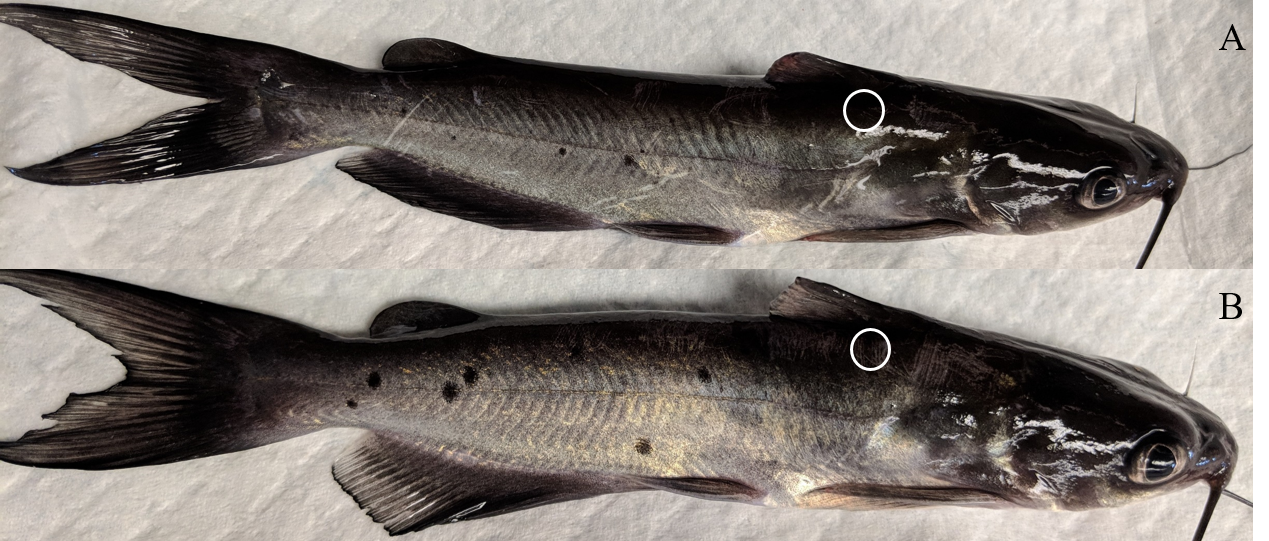


Figure S1. Channel catfish injected with Control (A) and planktonically-cultured vAh secreted proteins (B) 7 days post-injection. White circle denotes injection site. Fish injected with planktonic ECPs were indistinguishable from controls.
